# Emotional aspects and associated factors among pregnant teachers in the state education system of Minas Gerais, Brazil

**DOI:** 10.47626/1679-4435-2025-1443

**Published:** 2025-09-29

**Authors:** Lívia Gabriela de Souza Cardoso, Ana Carolina Queiroz de Oliveira, Rose Elizabeth Cabral Barbosa, Desirée Sant’Ana Haikal, Lucineia de Pinho

**Affiliations:** 1 Curso de Medicina, Centro Universitário FIPMoc, Montes Claros, MG, Brazil.; 2 Curso de Medicina, Universidade Estadual de Montes Claros (Unimontes), Montes Claros, MG, Brazil.; 3 Programa de Pós-Graduação em Ciências da Saúde, Unimontes, Montes Claros, MG, Brazil.; 4 Programa de Pós-Graduação em Cuidado Primário em Saúde, Unimontes, Montes Claros, MG, Brazil.

**Keywords:** anxiety, depression, mental disorders, pregnant women, school teachers, ansiedade, depressão, transtornos mentais, gestantes, professoras escolares

## Abstract

**Introduction:**

Female teachers are particularly vulnerable to developing depression and anxiety during pregnancy, and there are substantial research gaps regarding this specific group.

**Objectives:**

To investigate the prevalence and associated factors of anxiety and depression among pregnant teachers in the state public basic education system of Minas Gerais.

**Methods:**

This was an epidemiological web survey conducted from August 20 to September 11, 2020, using an online questionnaire sent to basic education teachers. The dependent variable was a diagnosis of depression and/or anxiety at any point in time. The analysis was restricted to data from pregnant teachers. Pearson’s chi-square test and Poisson regression with robust variance were applied.

**Results:**

Among the 232 pregnant teachers, 64.2% reported a diagnosis of depression and/or anxiety at some point. A higher prevalence of anxiety and/or depression was observed among those aged 36 years or older (prevalence ratio = 1.55; 95%CI 1.01-2.39) and those reporting mental health problems among family members (prevalence ratio = 2.06; 95%CI 1.31-3.22).

**Conclusions:**

The prevalence of anxiety and/or depression among pregnant teachers is high and is associated with being aged 36 years or older and reporting mental health problems among family members.

## Abstract (pt)

**Introdução:**

As professoras são especialmente vulneráveis ao desenvolvimento de depressão e ansiedade durante a gravidez, e existem lacunas substanciais na pesquisa voltada para esse grupo específico.

**Objetivos:**

Investigar a prevalência e os fatores associados à ansiedade e à depressão entre gestantes professoras da rede estadual de educação básica de Minas Gerais.

**Métodos:**

Trata-se de um inquérito epidemiológico do tipo *web survey,* realizado entre 20 de agosto e 11 de setembro de 2020, por meio de formulário on-line enviado aos professores da educação básica. A variável dependente foi diagnóstico de depressão e/ou de ansiedade em algum momento. A análise foi restrita aos dados das gestantes professoras, sendo aplicados o teste qui-quadrado de Pearson e a regressão de Poisson com variância robusta.

**Resultados:**

Entre as 232 gestantes professoras, 64,2% relataram diagnóstico de depressão e/ou ansiedade em algum momento. Observou-se maior prevalência de ansiedade e/ou depressão entre gestantes professoras com 36 anos ou mais (razão de prevalência = 1,55; intervalo de confiança de 95% 1,01-2,39) e que referiram problemas de saúde mental entre familiares (razão de prevalência = 2,06; intervalo de confiança de 95% 1,31-3,22).

**Conclusões:**

A prevalência de ansiedade e/ou depressão entre as gestantes professoras é elevada, sendo associada à idade igual ou superior a 36 anos e ao relato de problemas de saúde mental entre familiares.

## INTRODUCTION

Mental disorders are characterized by clinically significant disturbances in cognition, emotional regulation, or behavior, with individual psychological and biological factors — such as emotional and genetic traits — acting as risk factors.^1^ Among these mental health conditions, anxiety, marked by excessive fear and worry, and depression, which includes symptoms such as poor concentration, excessive guilt or low self-esteem, hopelessness about the future, thoughts of death or suicide, sleep disturbances, and changes in appetite or weight, stand out.^2^ Evidence in literature indicates that gender influences differing susceptibilities and exposures to specific mental health risks due to distinct biological processes and social relationships, making women more vulnerable to developing mental disorders.^3^

This vulnerability becomes more pronounced during the reproductive period, as mood modulation is mediated by gonadal hormones, which become unstable when interacting with other neuromodulators along the hypothalamic-pituitary-gonadal axis. This condition, typical of reproductive age, is strongly influenced by intense hormonal fluctuations and psychosocial factors, such as shifting social roles and low levels of community support. These aspects make the female body more vulnerable and more susceptible to the onset or relapse of mental disorders.^4,5^

Psychological changes in women can range from mild to severe, with those experiencing high-risk pregnancies being particularly susceptible to complications during this period.^6^ Sociodemographic, obstetric, clinical, and lifestyle factors can heighten stress during pregnancy and potentially predispose women to developing mental disorders. Factors such as teenage pregnancy, unplanned pregnancy, alcohol consumption during pregnancy, multiple gestations, lower income, being single, or having unstable relationships are associated with a higher likelihood of developing depressive and anxiety disorders.^7^ Thus, the high prevalence of depression during pregnancy underscores the importance of prioritizing and integrating mental health care into prenatal care, particularly in primary health care settings.

Mental health conditions during pregnancy can have significant impacts on maternal and child health, including an increased risk of miscarriage, preterm labor, low birth weight, lower Apgar scores, and postpartum depression. They may also negatively affect child development, contributing to behavioral problems and cognitive disorders after birth.^8^

An additional factor to consider is the occupation of the pregnant woman. Teaching, in particular, is a profession that exposes workers to high psychological strain due to multiple factors such as long working hours, low pay, lack of institutional support, heavy workloads, and challenges in relationships with students and parents.^9^ As a result, teachers face increased vulnerability to psychological disorders during pregnancy, compounded by the structural challenges inherent in the profession.

Thus, the multiple experiences faced during pregnancy make expectant mothers particularly vulnerable to developing depression and anxiety, with significant consequences for maternal and child health. Information on the prevalence of these mental disorders and their determinants can support effective strategies for prevention, early diagnosis, and intervention in prenatal care. However, there are substantial gaps in research focused on this specific group, especially in the context of Minas Gerais (MG). Therefore, the aim of this study was to investigate the prevalence of and factors associated with anxiety and depression among pregnant teachers in the public basic education system of MG, considering sociodemographic variables, occupational characteristics, and socio-affective aspects.

## METHODS

This study is part of the project “Health and working conditions among teachers in the state education system of Minas Gerais during the COVID-19 pandemic,” also known as the ProfSMoc Project – Minas Covid Stage. It is an epidemiological web survey conducted with basic education teachers working in state public schools. As an online survey, it followed the recommendations of the Checklist for Reporting Results of Internet E-Surveys (CHERRIES).^10^

The study population comprised approximately 90,000 teachers working in 3,441 state public schools, according to data provided by the State Department of Education of Minas Gerais (Secretaria de Estado de Educação de Minas Gerais, SEE-MG), based on the July 2020 payroll. The sample size was calculated using the formula for infinite populations. The minimum estimated sample was 2,564 teachers, considering a prevalence of 50%, a 95% confidence level, a 3% margin of error, a design effect (deff) of 2, and an additional 20% to account for possible losses.

The study included basic education teachers working in state schools who were pregnant at the time of data collection. Exclusion criteria were teachers who were not pregnant during the pandemic period up to data collection, those working in positions other than teaching (such as principals, coordinators, etc.), and those who answered “no” to the consent question regarding participation in the study.

Data collection took place from August 20 to September 11, 2020, using an online questionnaire hosted on the Google Forms® platform. The link to the form was sent by SEE-MG to the institutional email of all teachers in the state public system. To prevent automated responses, a reCAPTCHA with image-based tests was implemented. Participation was voluntary, and respondent anonymity was ensured. All questions in the form were mandatory to minimize missing data.

The dependent variable — diagnosis of depression and/or anxiety at any point — was obtained from responses to two questions: 1) Before the coronavirus pandemic, had you been diagnosed by a doctor with anxiety or depression? and 2) During the pandemic, have you been diagnosed by a doctor with anxiety or depression? The response options were no; yes, anxiety; yes, depression; yes, anxiety and depression. Teachers who answered “yes” to either or both questions were classified as POSITIVE for the outcome.

Independent variables included age (up to 35 years; 36 years or older), skin color (White; Black/Brown/Yellow/Indigenous), education level (postgraduate degree; no postgraduate degree), marital status (with a partner; without a partner), having other children (yes; no), monthly family income (up to 3 Brazilian minimum monthly salaries; 4 to 6 Brazilian minimum monthly salaries; 7 Brazilian minimum monthly salaries or more), having private health insurance (yes; no), reduced family income during the pandemic (yes; no), separation during the pandemic (yes; no), family arguments (yes; no), alcohol consumption among family members (yes; no), mental health problems among family members (yes; no), sexual harassment or violence during the pandemic (yes; no), increased domestic workload during the pandemic (yes; no), and teaching workload overload during the pandemic (yes; no).

All statistical analyses were performed using Stata software, version 13.0. Data were initially tabulated and analyzed using the distribution of relative and absolute frequencies of the study variables. Bivariate analyses were then conducted using Pearson’s chi-square test to estimate crude associations between variables. Variables with a significance level of 20% (p ≤ 0.20) in the bivariate analyses were selected for multiple analyses using Poisson regression with robust variance. Models were adjusted until only variables with a significance level of 5% (p ≤ 0.05) remained in the final model.

This study was conducted in accordance with Resolution 466/12 of the National Health Council, Ministry of Health. The project was reviewed and approved by the Research Ethics Committee of the Universidade Estadual de Montes Claros (Unimontes) in August 2020, under approval number 4.200.389. All participants received an informed consent form when accessing the survey.

## RESULTS

A total of 232 teachers were pregnant during the study period. Among them, 64.2% (n = 149) reported having been diagnosed with depression and/or anxiety at some point (before or during the pandemic), 45.3% (n = 105) frequently felt sad during the pandemic, 58.6% (n = 136) frequently felt anxious during the pandemic, and 13.4% (n = 31) used medication for sleep, depression, and/or anxiety.

Regarding sociodemographic characteristics, 60.3% were aged up to 35 years, 86.2% lived with a partner, 67.7% had children, and 54.3% had a monthly family income of up to 3 Brazilian minimum monthly salaries. As for occupational profile, 68.1% held a postgraduate degree, and 74.6% reported teaching workload overload during the pandemic. The remaining data, related to socio-affective aspects, are presented in Table 1.

**Table 1:** Descriptive and bivariate analysis of sociodemographic characteristics, occupational profile, and socio-affective aspects of pregnant basic education teachers in the state public school system (n = 232), ProfSMoc – Minas Covid Stage, Minas Gerais, 2020

Variables	n	%	p (%)	p-value*
Sociodemographic characteristics				
Age (years)				0.011
Up to 35	140	60.3	29.3	
36 or older	92	39.7	45.7	
Skin color				0.367
White	107	46.1	32.7	
Black/Brown/Yellow/Indigenous	125	53.9	38.4	
Marital status				0.827
Without partner	32	13.8	37.5	
With partner	200	86.2	35.5	
Other children				0.157
Yes	157	67.7	38.9	
No	75	32.3	29.3	
Monthly family income (Brazilian minimum monthly salaries)				0.309
Up to 3	126	54.3	33.3	
4 to 6	91	39.2	36.3	
7 or more	15	6.5	53.3	
Reduced family income during the pandemic				0.090
No	131	56.5	40.5	
Yes	101	43.5	29.7	
Has private health insurance				0.156
Yes	60	25.9	43.3	
No	172	74.1	33.1	
Occupational profile				
Teaching workload overload during the pandemic				0.728
No	59	25.4	33.9	
Yes	173	74.6	36.4	
Education level				0.108
With postgraduate degree	158	68.1	39.2	
Without postgraduate degree	74	31.9	28.4	
Socio-affective aspects				
Family arguments				0.208
No	194	83.6	34.0	
Yes	38	16.4	44.7	
Alcohol consumption among family members				0.008
No	154	66.4	29.9	
Yes	78	33.6	47.4	
Mental health problems among family members				0.000
No	182	78.5	29.1	
Yes	50	21.5	60.0	
Experienced sexual harassment or violence during the pandemic				0.337
No	222	95.7	35.1	
Yes	10	4.3	50.0	
Separation during the pandemic				0.049
No	223	96.1	34.5	
Yes	9	3.9	66.7	
Increased domestic workload during the pandemic				0.109
No	62	26.7	27.4	
Yes	170	73.3	38.8	

* Pearson’s chi-square test

The results of the bivariate analysis between a diagnosis of depression and/or anxiety at any point and sociodemographic, occupational, and socio-affective variables are presented in Table 1. Variables with a significance level of 20% (p ≤ 0.20) were selected for multiple analysis.

The variables that remained in the multiple model after adjustments are shown in Table 2. Regarding sociodemographic characteristics, the prevalence of anxiety and/or depression among pregnant teachers was higher among those aged 36 years or older (prevalence ratio [PR] = 1.55). As for socio-affective aspects, a higher prevalence of anxiety and/or depression was observed among those who reported mental health problems among family members (PR = 2.06).

**Table 2:** Multiple analysis, diagnosis of anxiety and/or depression, ProfSMoc – Minas Covid Stage, Minas Gerais, 2020 (n = 232)

Variables	PR (95%CI)	p-value
Age (years)		
Up to 35	1.00	
36 or older	1.55 (1.01-2.39)	0.044
Mental health problems among family members		
No	1.00	
Yes	2.06 (1.31-3.22)	0.002

PR = prevalence ratio.

## DISCUSSION

This study revealed a high prevalence of neuropsychiatric disorders among pregnant teachers in the state public school system of MG, with 64.2% (n = 149) of the 232 participants reporting a diagnosis of depression and/or anxiety at some point (before or during the pandemic).

Internationally, the prevalence of depression and anxiety during pregnancy is also well documented. In Asia, for instance, a study conducted in Jakarta, Indonesia, found that 59.7% of pregnant women showed symptoms of depression.^11^ Similarly, studies in Saudi Arabia and Thailand reported high rates of neuropsychiatric disorders, with more than 25.0% of women in both studies experiencing depression, and anxiety prevalence reaching 23.6% among pregnant women in Saudi Arabia.^12,13^ Epidemiological data from Egypt indicated a marked prevalence of prenatal depression, reporting depressive symptom rates of 13.2% during pregnancy and 8.1% postpartum, based on the Edinburgh Postnatal Depression Scale.^14^ Among South African women, the prevalence of prenatal depression and anxiety was 27% and 15.2%, respectively.^15^

In Europe, a study conducted in Slovenia showed that 21.7% of pregnant women were identified as having high depressive symptomatology, 15.7% reported high state anxiety, and 12.5% presented high trait anxiety.^16^ In Australia, the prevalence of prenatal and postnatal depressive symptoms was 6.2% and 3.3%, respectively.^17^ In North America, a study from Mexico found that the prevalence of depressive symptoms was 16.6% in the prenatal period, 17.1% at 6 weeks of gestation, and 20.0% at 6 months postpartum.^18^

This scenario is consistent with the Brazilian context, where studies from different regions also point to a high prevalence of mental disorders during pregnancy. For example, research conducted in Ribeirão Preto, São Paulo, indicated that the incidence of prenatal depression in high-risk pregnancies ranged from 12.5% to 44.2%.^19^ Another study with similar objectives, conducted among women receiving primary health care in Cruzeiro do Sul, Acre, reported that the prevalence of common mental disorders during pregnancy was 36.2% and 24.5% in the first 2 assessments of the Maternal and Child Health and Nutrition in Acre, Brazil (MINA-Brazil) study.^7^

Furthermore, data from a study including 153 women in their third trimester of pregnancy (≥ 27 weeks) living in a municipality in the countryside of Rio Grande do Sul showed that, regarding emotional, physical, and psychological occurrences, 32% of participants reported at least 1 such occurrence, 26.1% reported experiencing stressful or traumatic events, and 34% reported emotional or physical complications.^20^

According to the International Labour Organization (ILO), teaching is classified as a high-risk occupation, ranking second worldwide among professions most susceptible to the development of occupational diseases.^21^ Teachers’ working conditions — including the devaluation of their role in society, long working hours, low salaries, and lack of recognition — have a direct impact on their mental health.^9^ Additionally, female teachers report a greater perception of workplace pressure compared to male teachers, due to the normalization of the belief that work performed by women is of lower qualification.^22^

The workload burden is further intensified by the greater volume of social demands and domestic responsibilities.^9,22^ Teachers’ mental health significantly affects the quality of education and the functioning of the school system, potentially leading to socioeconomic consequences such as increased organizational costs, staff turnover, absenteeism, and reduced productivity.^9^

Maternal mental health problems, such as depression and anxiety, can affect not only the mother but also the baby, both in the short and long term. Findings from a study conducted in a tertiary medical center showed that women with anxiety disorders are at greater risk of adverse perinatal outcomes, such as preterm birth, hypertensive disorders, and cesarean delivery, and that their children have a higher prevalence of neuropsychiatric hospitalization.^8^ These repercussions are even more critical when they occur in professionals who also carry caregiving responsibilities, such as teachers, highlighting the need for targeted mental health interventions for this population.

The diagnosis of anxiety and/or depression was more frequent among pregnant teachers aged 36 years or older. This result is consistent with a prospective cohort study of all women with singleton live births in Sweden from 1997 to 2008, which found that the risk of developing postpartum depression (PPD) was higher among women over 35 years of age compared with those aged 25-29 years.^23^ A cross-sectional study involving 292 primiparous mothers at a maternal and child hospital in southern China reported that younger maternal age was a protective factor against prenatal depressive symptoms.^24^ In Brazil, a cohort study conducted in the southern region found that pregnant women aged 35 years or older were 36% more likely to present prenatal depressive symptoms compared with those aged 20 years or younger, reinforcing the findings of the present study.^25^

Pregnancy at 35 years of age or older is considered high risk due to the greater likelihood of adverse outcomes. Hypertensive syndromes, gestational diabetes mellitus, chromosomal abnormalities, congenital malformations, prematurity, low birth weight, low Apgar score, admission to neonatal intensive care, and perinatal mortality are all associated with advanced maternal age.^26^ Concerns about the progression of the current pregnancy and the possibility of complications make expectant mothers more vulnerable to feelings of fear and anxiety, contributing to the development of mental disorders during the pregnancy-postpartum period.^27^ Furthermore, the occurrence of depression and/or anxiety during pregnancy can negatively affect the mother-child relationship, as it may reduce maternal acceptance of the child and shorten breastfeeding duration, potentially leading to delays in child development.^5,6^

In the present study, mental health problems among family members were significantly associated with a higher prevalence of anxiety and/or depression in pregnant teachers. This finding is consistent with international evidence. A cohort study conducted with 55 pregnant women and 144 postpartum women in Italy, at the Perinatal Psychology Outpatient Service in Ancona, found that a positive family psychiatric history is a strong predictor of perinatal depression.^28^ Similarly, a systematic review and meta-analysis that included 24 cohort studies and 2 case-control studies, totaling 100,877 postpartum women, identified an almost 2-fold higher risk of developing postpartum depression in mothers with a family history of psychiatric disorders.^29^ Maternal history of prenatal depression is also linked to significant adverse effects on offspring health, as daughters of women with such a history have a 3-fold higher risk of developing depression during pregnancy compared with those without a positive family history.^30^

From a clinical perspective, this study is relevant in establishing factors that influence the occurrence of depression and/or anxiety during pregnancy. Understanding these factors is crucial for organizing and implementing measures for the prevention, identification, follow-up, and management of these neuropsychiatric conditions in prenatal care. This reinforces the importance of epidemiological analysis of the population to guide the structuring of health services and the training of professionals, aiming to identify pregnant women at risk of developing mental disorders and prevent adverse outcomes for both mother and child.^5,6^

Some limitations of this study must be considered. As an epidemiological web survey, there is a potential selection bias since its reliance on internet access. Responses were self-reported, which may be influenced by recall bias. Nevertheless, the findings allow for discussion on a topic that remains underexplored in the Brazilian context, particularly in MG. Moreover, this population-based survey provided relevant epidemiological evidence for future research and for promoting pregnant women’s health. It was conducted with methodological rigor and a substantial sample size, which strengthened the associations observed.

This study contributes to the understanding of mental health issues in a strategic segment of the workforce: women, pregnant women, and public-school educators. The triple vulnerability of these women calls for specific public policies, care strategies tailored to the realities of teaching work, and the recognition of teachers’ societal role. The data presented offer valuable input for planning mental health actions for pregnant women in adverse occupational contexts. It also reinforces the importance of high-quality prenatal follow-up, particularly for women in professions with a high emotional workload.

## Conclusions

The prevalence of depression and/or anxiety among pregnant teachers in the state public basic education system of MG was high. Age 36 years or older and the presence of mental health problems among family members were significantly associated with a diagnosis of depression and/or anxiety during pregnancy in this population.
